# Diet restriction in migraine, based on IgG against foods: A clinical double-blind, randomised, cross-over trial

**DOI:** 10.1177/0333102410361404

**Published:** 2010-07

**Authors:** Kadriye Alpay, Mustafa Ertaş, Elif Kocasoy Orhan, Didem Kanca Üstay, Camille Lieners, Betül Baykan

**Affiliations:** 1 Istanbul Faculty of Medicine, Department of Neurology, Istanbul, Turkey.; 2 Vivitro Ltd, Istanbul, Turkey.; 3 Invitalab, Neuss, Germany.

**Keywords:** migraine, food, diet, IgG, trigger

## Abstract

*Introduction:* It is well-known that specific foods trigger migraine attacks in some patients. We aimed to investigate the effect of diet restriction, based on IgG antibodies against food antigens on the course of migraine attacks in this randomised, double blind, cross-over, headache-diary based trial on 30 patients diagnosed with migraine without aura.

*Methods:* Following a 6-week baseline, IgG antibodies against 266 food antigens were detected by ELISA. Then, the patients were randomised to a 6-week diet either excluding or including specific foods with raised IgG antibodies, individually. Following a 2-week diet-free interval after the first diet period, the same patients were given the opposite 6-week diet (provocation diet following elimination diet or vice versa). Patients and their physicians were blinded to IgG test results and the type of diet (provocation or elimination). Primary parameters were number of headache days and migraine attack count. Of 30 patients, 28 were female and 2 were male, aged 19–52 years (mean, 35 ± 10 years).

*Results:* The average count of reactions with abnormally high titre was 24 ± 11 against 266 foods. Compared to baseline, there was a statistically significant reduction in the number of headache days (from 10.5 ± 4.4 to 7.5 ± 3.7; *P* < 0.001) and number of migraine attacks (from 9.0 ± 4.4 to 6.2 ± 3.8; *P* < 0.001) in the elimination diet period.

*Conclusion:* This is the first randomised, cross-over study in migraineurs, showing that diet restriction based on IgG antibodies is an effective strategy in reducing the frequency of migraine attacks.

## Introduction

The exact pathophysiology of migraine is still unclear. Besides different genetic mutations, there is evidence of a profound role of meningeal inflammation in migraine pathogenesis (1,2). Environmental trigger factors are thought to play an important role. Many contributing factors may trigger the occurrence of migraine attacks and food is one of the most well-known (3–8). These, however, as with most elements of migraine, need to be individualised to the patient with migraine.

Since the 1930s, hidden food allergy has been suspected to be linked to migraine. Several studies showed significant improvement when patients were put on an elimination diet (9–14). IgE-specific food allergy has been shown to be related with migraine supported by the success of individualised diet in controlling migraine attacks (4,15). Non-IgE antibody mediated mechanisms have also been proposed in food allergy (16). Aljada et al. (17) provided evidence for the pro-inflammatory effect of food intake. IgG antibodies against food antigens have been found to be correlated with inflammation and intima media thickness in obese juveniles (18). Several studies reported significant improvement in irritable bowel syndrome (IBS) by food elimination based on IgG antibodies against to food antigens (19–22). Rees et al. (23) showed a beneficial effect of a diet guided by IgG antibodies to food in migraine patients. Recently, Arroyave Hernandez et al. (24) reported preliminary evidence that IgG-based elimination diets successfully controlled the migraine without need of medication.

Some foods (such as cheese, chocolate or wine) are thought to be one of the well-known reasons triggering of migraine attacks according to consistent reports from the patients. It has been reported that diet with low-fat intake could reduce the headache frequency and intensity (25). On the other hand, some additives (such as triclorogalactosucrose or aspartame) may trigger attacks in some migraineurs (4,26–29). However, it is neither easy nor very useful to organise routine diet according to robust protocols for many patients (3,30). All this indicates that there is a need for an individualised approach of the diet to relieve migraine. One has to distinguish between inflammation-induced migraine and migraine caused by food via other mechanisms such as histamine-induced vasodilatation.

IgG could be one of the markers to identify food which causes inflammation and could cause migraine attacks in predisposed individuals.

In this study, we aimed to test the beneficial effect of diet based on specific total IgG antibodies (subclasses 1–4) against 266 food antigens in controlling migraine in a double-blind, randomised, controlled, cross-over clinical trial.

## Subjects and methods

### Experimental protocol

This study was designed as a double-blind, randomised, controlled, cross-over clinical trial (31). After the approval of the hospital ethics committee, patients giving their written informed consent were recruited from headache out-patient clinic with the diagnosis of migraine without aura according to the criteria of the International Classification of Headache Disorders, 2nd edition (32). For inclusion in the study, the patients should: (i) have had at least 4 attacks and 4 headache days per month within the last months; (ii) be aged 18–55 years; (iii) be treated with acute attack medications only or with preventive medications unchanged at least for 3 months; and (iv) be able to understand and co-operate with the needs of the study and the diet. The patients with suspected or clear-cut medication overuse, pure menstrual migraine or any other associated headache disorder were excluded.

**Figure 1.**
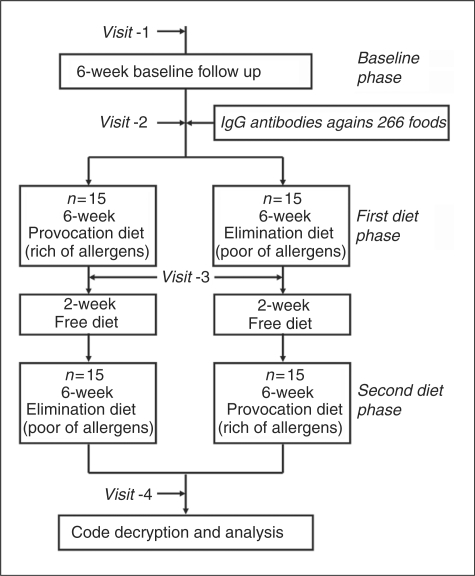
Cross-over design of the study.

The study consisted of three main phases – baseline phase, first diet phase and second diet phase (Figure 1). In all phases, patients were asked not to change the dosages of their preventive medications if they were using any. The patients visited the same headache physician (first author, blinded to antibody test results and the order of the patient’s diet phases) during the whole study. At the first visit (Visit-1), the eligible patients recruited for the study were evaluated and asked to complete a headache diary for 6 weeks. The diary included headache attack frequency, headache days, attack duration in hours, attack severity in visual analogue scale (VAS) and medication information. During this 6-week baseline phase, the patients were followed in their usual daily diet. At the second visit (Visit-2) at the end of this 6-week baseline phase, the patients returned their diaries for evaluation and gave venous blood samples for detection of IgG antibodies against 266 food antigens (see Appendix 1) as described below. Then, the patients were allocated to one of the two 6-week diets based on a randomisation schedule either excluding (elimination diet) or including (provocation diet) specific foods with abnormally raised IgG antibodies, as described below. They were also asked to fill in a headache diary for 6 weeks. In this ‘first diet phase’, half of the patients were randomised to elimination diet and other half maintained provocation diet for 6 weeks. Neither the patients nor the headache physician knew if the diet was for elimination or provocation as well as IgG antibody test results. At the third visit (Visit-3) at the end of the ‘first diet phase’, the patients returned their diaries for evaluation. Then, the patients were allowed to return to their usual diets for 2 weeks without keeping diary. In ‘second diet phase’ following this 2-week diet-free interval, the patients who were on elimination diet in the ‘first diet phase’ were given provocation diet for 6 weeks and vice versa. They were asked to fill a headache diary for 6 weeks during the second diet phase. At the fourth visit (Visit-4) following the ‘second diet phase’, diaries were recollected and the diet codes were broken. Patients were informed about their results and the order of the types of their diets.

### IgG antibody detection against food antigens

IgG antibodies against 266 food antigens were detected using a commercially available enzyme linked immunosorbant assay (ELISA) test (ImuPro 300 test; Evomed/R-Biopharm AG, Darmstadt, Germany), previously used by Wilders-Truschnig et al. (18) IgG calibration was performed against the international reference material 1st WHO IRP 67/86 for human IgG. Quantitative measurements are reported in mg/l. Detection limit was 2.5 g/l, normalised cut-off value was 7.5 mg/l, according to the validation protocol provided by the manufacturer. All values above 7.5 mg/l were considered as positive reaction to the corresponding food. Positive reaction was graded as: ‘low’ for titres between 7.50–12.50 mg/l; ‘moderate’ for 12.51–20 mg/l; ‘high’ for 20.1–50 mg/l; and ‘very high’ for 50.1–200 mg/l. Since IgG antibodies against food disappear within 3 months to 2 years depending upon initial titre, antibody detection was not repeated after the diet phases which were not long enough to see any change in antibody titres.

### Diet preparation

Diets were arranged according to the IgG antibody results; eating habits for individual patients were taken into account and patients were educated about keeping diet by a dietician (DKU, co-author). Diets were assembled by this dietician, trying to offer a nutritionally balanced diet. The elimination diet consisted of a defined panel of IgG-negative food, while the provocation diet consisted in a panel of IgG-positive food and IgG-negative food necessary to comply with a balanced diet. Both elimination diet and provocation diet did not differ in calorie content. Patients were not allowed to eat any other food as specified by the dietician. In both diet phases, patients were never forced to eat or avoid from certain foods to protect the blindness of patients and their physician for the type of diet phase (elimination or provocation). Instead, they were asked to follow their specially arranged diet list exactly and not to consume any other food in any diet phase.

### Statistical analysis

The primary parameters used for comparisons were number of headache days, migraine attack count, mean attack duration, median attack severity (in VAS score from 0–10), number of migraine attacks with acute medication and total medication intake within 6-week periods of each three phases. Statistical analyses included parametric tests to compare means (paired and unpaired two sample *t*-test), non-parametric tests to compare medians (Wilcoxon test and Mann–Whitney test) and 2 × 2 chi-square test. For comparisons between baseline phase and provocation phase or between baseline phase and elimination phase or between provocation phase and elimination phase, both paired *t*-tests (parametric test and Wilcoxon signed test as non-parametric test) were used. For comparisons between patients whose first diet phase was elimination diet and patients whose second diet phase was elimination diet (to test the ‘period effect’), both unpaired *t*-tests (parametric test and Mann–Whitney unpaired test as a non-parametric test) were used.

## Results

A total of 35 patients were recruited for the study. In the baseline phase and the first diet phase, five patients withdrew from the study for different reasons, such as moving to another city, unwillingness to maintain diet or skipping the visit. The remaining 30 patients fully completed the study. Of these 30 patients, 28 were females and 2 were males. Ages varied from 19 years to 52 years (mean, 35 ± 10 years). Their mean migraine duration was 13 ± 9 years (range, 1–30 years). All 30 patients were using acute attack medication for migraine attacks and 15 were also using preventive medication. From the IgG antibody tests against to 266 food allergens, mean reaction (abnormally high titre) count was 24 ± 11 (7–47 reactions in individual patients). Of the total 732 reactions, 297 (40%) were low, 337 (46%) were moderate, 70 (10%) were high and 28 (4%) were very high graded. The food categories are listed in Table 1 from the most frequent IgG positivity to least.

**Table 1.** The food categories from most frequent IgG positivity to least

	Number of patients with positive test result (*n* = 30)
Spices	27
Seeds and nuts	24
Seafood	24
Starch	22
Food additives	21
Vegetables	21
Cheese	20
Fruits	20
Sugar products	20
Other additives	14
Eggs	14
Milk and milk products	14
Infusions	13
Salads	10
Mushrooms	9
Yeast	5
Meat	5

The parameters of headache and medication are shown in Tables 2 and 3. In the elimination diet period compared to both baseline period and provocation diet period, there was a statistically significant reduction in attack count, number of headache days, number of attacks with acute medication and total medication intake whilst no significant change occurred between the provocation diet period and the baseline period (Table 2). However, attack severity and attack duration did not change significantly between all three phases (Table 2). By comparing percentage differences from baseline, in the elimination diet period, attack count, number of headache days, number of attacks with acute medication and total medication intake showed significant reduction compared to the provocation diet period (Table 3). To test the effect of order of diet, patients were divided to two subgroups: 15 patients with elimination diet in the ‘first diet phase’ and 15 patients with elimination diet in the ‘second diet phase’. We calculated the differences (in both real numbers and percentage differences) between the elimination diet period and the baseline, provocation diet period and baseline and elimination diet period and provocation diet period for attack count, number of headache days, number of attack with acute medication, total medication intake, median attack severity and mean attack duration for each subgroup. In none of these parameters, did the two subgroups show any significant difference in any of these comparisons, indicating that, whether the elimination diet was in the first diet phase or second diet phase, the order of elimination diet period had no significant effect on the outcome (Table 4). Similar calculations were made for preventive medicine usage and there was no significant change in any parameters between preventive medicine users (*n* = 15) and non-users (*n* = 15).

**Table 2.** Headache and medication parameters during study phases

	Phases, each of 6 weeks ± SD (95% CI lower; upper limits)		
Parameters	Baseline	Provocation diet	Elimination diet
Attack count	8.97 ± 4.4 (7.32; 10.61)	8.13 ± 4.6 (6.43; 9.83)	*6.17 ± 3.8 (4.67; 7.58)
Number of headache days	10.53 ± 4.4 (8.90; 12.17)	10.20 ± 5.5 (8.15; 12.25)	*7.47 ± 3.7 (6.08; 8.85)
Number of attacks with acute medication	6.73 ± 2.9 (5.65; 7.81)	6.53 ± 4.0 (5.05; 8.01)	^†^4.90 ± 3.2 (3.69; 6.11)
Total medication intake (tablets)	11.37 ± 7.4 (8.61; 14.13)	10.57 ± 7.7 (7.69; 13.44)	^‡^7.77 ± 5.7 (5.65; 9.89)
Median attack severity (VAS)	6.02 ± 1.6 (5.42; 6.62)	6.07 ± 1.6 (5.47; 6.66)	6.07 ± 1.6 (5.60; 6.77)
Mean attack duration (hours)	11.39 ± 5.6 (9.30; 13.48)	12.53 ± 6.7 (10.04; 15.03)	12.53 ± 6.7 (9.57; 15.14)

*P < 0.001/*P* < 0.001; ^†^*P* < 0.001/*P* = 0.001; ^‡^*P* = 0.002/*P* = 0.001 in Paired *t*-test/Wilcoxon signed test (with 95% confidence intervals) comparing elimination and baseline diet phases (differences are statistically significant).

**Table 3.** Percentage difference of parameters from baseline period

Parameters	Provocation diet* (% difference) mean ± SD (95% CI limits^†^)	Elimination diet* (% difference) mean ± SD (95% CI limits^†^)	*P*-values^‡^ paired *t*-test/ Wilcoxon test
Attack count	−7.81 ± 37.6 (−21.85; 6.23)	−29.11 ± 26.0 (−38.83; −19.39)	*P* = 0.006/0.004
Number of headache days	−2.77 ± 35.2 (−15.93; 10.38)	−25.68 ± 30.9 (−37.22; −14.13)	*P* = 0.006/0.006
Number of attacks with acute medication	3.49 ± 66.4 (−21.30; 28.29)	−20.50 ± 54.2 (−40.74; −0.27)	*P* = 0.006/0.002
Total medication intake (tablets)	7.34 ± 67.0 (−17.68; 32.35)	−16.33 ± 56.6 (−37.41; 4.75)	*P* = 0.006/0.006
Median attack severity (VAS)	5.12 ± 33.3 (−7.33; 17.56)	8.15 ± 42.0 (−7.53; 23.82)	NS (*P* = 0.348/0.460)
Mean attack duration (h)	25.19 ± 81.6 (−5.29; 55.67)	12.79 ± 53.3 (−7.10; 32.68)	NS (*P* = 0.442/0.738)

*Percentage difference from baseline period; ^†^(lower; upper) limits of 95% confidence interval; ^‡^*P*-values with 95% confidence interval; NS, not significant.

**Table 4.** Percentage difference of elimination diet parameters from baseline period between the groups starting with elimination diet and starting with provocation diet

	Elimination diet		
Parameters	At 1. Diet* (% difference^†^) median value (percentiles^‡^) *n* = 15	At 2. Diet** (% difference) median value (percentiles) *n* = 15	*P*-value (Mann–Whitney unpaired test)
Attack count	−37.5 (−55.6; −20.0)	−30.0 (−35.0; −16.7)	NS (*P* = 0.367)
Number of headache days	−30.0 (−50.0; 0.0)	−33.3 (−46.2; 0.0)	NS (*P* =0.902)
Number of attacks with acute medication	−37.5 (−55.6; 0.0)	−30.0 (−50.0; 0.0)	NS (*P* = 0.653)
Total medication intake (tablets)	−25.0 (−65.2; 20.0)	−18.2 (−37.9; 0.0)	NS (*P* = 0.539)
Median attack severity (VAS)	0.0 (0.0; 14.3)	0.0 (−20.0; 14.3)	NS (*P* = 0.512)
Mean attack duration (h)	10.9 (−29.0; 74.9)	3.7 (−27.3; 13.0)	NS (*P* = 0.267)

*Patients starting with elimination diet; **patients starting with provocation diet; ^†^ percentage difference from baseline period; ^‡^(25%; 75%) percentiles.

NS, not significant in Mann-Whitney test with 95% confidence interval.

We also calculated if the patient showed at least a 30% reduction and at least a 50% reduction for attack count and number of headache days using elimination diet compared to provocation diet and to the baseline period. In comparison between elimination and baseline phases, for parameters of the number of headache days and attack count, reduction was ≥30% in 16 (53%) and 16 (53%) patients, respectively, and reduction was ≥50% in 7 (23%) and 6 (20%) patients, respectively. In comparison between elimination and provocation phases, for the number of headache days and attack count, reduction was ≥30% in 15 (50%) and 12 (40%) patients, respectively, and reduction was ≥50% in 6 (20%) and 4 (13%) patients, respectively.

## Discussion

The concept that food may trigger some symptoms creates an increasing pressure on the healthcare system to investigate possible causal relationships between food intake and specific diseases. In the case of migraine, it seems evident that food is not the primary cause but, via different mechanisms, is able to induce or aggravate migraine attacks. In some individuals, the consumption of chocolate or red wine is enough to provoke an attack; whereas, in others, a combination of food is required, even sometimes with food which has never been related to migraine for other migraineurs. However, it is neither easy nor very useful to organise routine diet according to robust protocols for many patients (3,30). All this indicates that there is a need for an individualised approach to diet to relieve migraine.

A recent study addressed a relationship between IgG antibodies against food antigens and systemic inflammation measured by C-reactive protein (CRP) in obese juvenile patients (18). Obesity was also shown as an important risk factor in the development of chronic daily headache and chronic migraine (33,34). The therapeutic potential of dietary elimination on the basis of the presence of IgG antibodies against food in patients with IBS has been investigated. Patients were randomised to receive either a diet excluding all foods to which they had raised IgG antibodies or a sham diet excluding the same number of foods but not those to which they had antibodies (19). The true diet resulted in a statistically significant reduction in symptom scores compared to the sham diet and the authors concluded that this technique is worthy of further clinical research in IBS.

There is growing evidence that inflammation plays an important role in the pathogenesis of migraine (2). The calcitonin gene-related peptide (CGRP), and nitric oxide (NO) may participate in immune and inflammatory responses. Some patients report that certain foods only trigger migraine in conjunction with stress or extended physical exercise. Both conditions, recognised as triggers of migraine, cause the release of pro-inflammatory cytokines. In these cases, inflammation caused by food could create the pro-inflammatory milieu necessary for the induction of migraine by other triggers. If we focus on inflammation induced by food, a specific marker is needed. All IgG subclasses, except IgG4 lead to an inflammatory response when in contact with the respective antigen. Determination of specific IgG to a large number of foods is an ideal tool to detect individually suspected food and enables a modification of nutritional habits in order to prevent chronic inflammation and onset of migraine in sensitised patients. Susceptibility to other triggers such as histamine, caused by impaired detoxification by low activity of di-amino-oxidase, may play an additional role and could be considered in the proposed diet.

A recent study from Mexico investigated 56 patients with recurrent attacks of migraine (at least once a month) and 56 control subjects without migraine and measured their allergen-specific IgG against 108 food allergens by enzyme immunoassay (24). The authors reported that there was a statistically significant difference in the number of positive results for IgG food allergens between patients and controls and the elimination diet successfully controls the migraine without need of medication. As distinct from this study, we used a randomised, cross-over design with clear definition of diagnostic and follow-up criteria and study set-up. We used a baseline period and provocative diet periods to compare elimination diet period of the same individuals as their own controls instead of using other healthy volunteers.

Our data confirm the importance of determination of specific IgG antibodies against food antigens for prevention and cure of food-induced migraine attacks, leading to lower drug consumption, fewer adverse drug reactions and fewer days with migraine.

## Conclusions

Diet restriction based on IgG antibodies might be an effective strategy in reducing the frequency of migraine attacks and could be implemented for therapy-resistant patient. However, because of the small sample size of this study, caution is required while translating these results into daily practice. Further research is also needed to determine the mechanism of IgG positive, food-induced migraine and its relation to other triggers.

## Appendix 1

### Full list of 266 food allergens tested

**Milk**
Milk (cow)
  Goat: milk and cheese

**Milk products**
Buttermilk
  Cottage cheese
  Yogurt
  Whey
  Curd cheese
  Camembert
  Edam cheese
  Emmenthal cheese
  Gouda cheese
  Leerdamer cheese
  Mozzarella
  Parmesan
  Ricotta
  Roquefort
  Processed cheese
  Tilsit cheese
  Sheep cheese

**Food additives**
Benzoic acid (E211)
  Citric acid (E330)
  Sorbic acid (E200)
  Agar-Agar (E406)
  Carrageen (E407)
  Gelatine
  Guar flour (E412)
  Pectin (E440)
  Traganth (E413)
  Amarant (E123)
  Azorubin (E122)
  Quinoline yellow (E104)
  Cochinella (E120)
  Erytrosin (E127)
  Yellow Orange FCF (E110)
  Curcumin (E100)
  Tartrazine (E102)
  Glutamate (E621)
                              *Aspergillus niger*
  Starch
  Spelt
  Barley
  Gluten
  Unripe spelt grain
  Oat
  Kamut
  Rye
  Wheat
  Amaranth
  Broad bean
  Buckwheat
  Millet
  Potato
  Chickpeas
  Lentil
  Maize, sweet corn
  Quinoa
  Rice
  Soy bean
  Tapioca, cassava

**Infusions**
Valerian
  Nettle
  Tea, green
  Rose hip
  Hibiscus leaves
  Coffee
  Chamomile
  Lime blossoms
  Rooibus tea
  Tea, black
  Hawthorn

**Other additives**
Aloe Vera
  Rape-seed oil
  Tannin
  Eggs
  Yellow chicken egg
  Chicken egg white

**Sugar products**
Agave nectar
  Maple syrup
  Aspartame (E951)
  Carob
  Honey
  Coconut
  Malt
  Cane sugar

**Spices**
Aniseed
  Basil
  Savory
  Cayenne pepper
  Chili
  Candied lemon peel
  Curry powder
  Dill
  Vervain
  Hop
  Ginger
  Cardamom
  Chervil
  Garlic
  Coriander
  Caraway
  Lavender
  Lovage
  Bay leaf
  Marjoram
  Horseradish
  Nutmeg
  Clove
  Oregano
  Paprika
  Parsley
  Parsley root
  Peppermint
  Allspice
  Rosemary
  Saffron
  Sage
  Chive
  Pepper, black
  Mustard seed
  Thyme
  Vanilla
  Juniper berry
  Pepper, white
  Cinnamon
  Lemon balm

**Meat**
Veal
  Lamb
  Beef
  Pork
  Duck
  Goose
  Chicken
  Ostrich meat
  Turkey hen
  Quail
  Rabbit
  Deer
  Hare
  Roe deer
  Wild boar

**Seafood**
Eel
  Anchovy
  Trout
  Shark
  Sole
  Herring
  Cod, codling
  Carp
  Salmon
  Mackerel
  Ocean perch
  Sardine
  Haddock
  Plaice
  Swordfish
  Tuna fish
  Zander
  Oysters
  Blue mussels
  Squid
  Shrimp, prawn
  Lobster
  Crayfish

**Fruits**
Pineapple
  Apple
  Apricot
  Banana
  Pear
  Blueberry
  Blackberry
  Strawberry
  Fig
  Raspberry
  Honeydew melon
  Cherry
  Kiwi
  Lychee
  Mandarin
  Mango
  Yellow plum
  Nectarine
  Orange
  Grapefruit
  Papaya
  Peach
  Plum
  Cranberry
  Quince
  Sea buckthorn
  Currant
  Gooseberry
  Grape
  Watermelon
  Lemon
  Sugar melon
  Chestnut
  Date
  Raisins
  Avocado
  Olive

**Seeds and nuts**
Cashew kernels
  Peanut
  Hazelnut
  Coconut
  Pumpkin seeds
  Linseed
  Almond
  Poppy seeds
  Brazil nut
  Pine nut
  Pistachio
  Sesame
  Sunflower seed
  Walnut

**Vegetables**
Artichoke
  Aubergine
  Bamboo shoots
  Cauliflower
  Beans, yellow
  Green bean
  Broccoli
  Chinese cabbage
  Green pea
  Fennel
  Savoy cabbage
  Cucumber
  Carrots
  Rutabaga
  Pumpkin
  Leek
  Chard, beet greens
  Green gram
  Sweet pepper
  Radish red
  Radish black
  Rhubarb
  Brussels sprouts
  Beetroot
  Red cabbage
  Shallot
  Black salsify
  Celeriac, knob celery
  Asparagus
  Spinach
  Tomato
  White cabbage
  Kale, curled kale
  Courgette
  Onion
  Salads
  Watercress
  Chicory
  Iceberg lettuce
  Endive
  Butterhead lettuce
  Head lettuce
  Dandelion
  Radicchio
  Romaine/Cos lettuce
  Rocket

**Mushrooms**
Oyster mushrooms
  Meadow mushrooms
  Bay boletus
  Chanterelle
  Shiitake
  Cep (boletus)

**Yeast**
Yeast
  Baking powder
